# Understanding Energy Expenditure: An Approach to Improving Activities of Daily Living in Huntington’s Disease

**DOI:** 10.3390/jcm15134999

**Published:** 2026-06-26

**Authors:** Lucía Simón-Vicente, Jéssica Rivadeneyra-Posadas, María Soto-Célix, Javier Raya-González, Alejandro Rodríguez-Fernández, Daniel Castillo-Alvira, Esther Cubo

**Affiliations:** 1 Health Science Department, University of Burgos, 09002 Burgos, Spain; lsvicente@ubu.es; 2 Research Unit, Burgos University Hospital, 09006 Burgos, Spain; 3 Faculty of Health Sciences, Universidad Isabel I, 09003 Burgos, Spain; msotocelix@gmail.com; 4 Endocrinología y Nutrición, Servicio Medicina Interna, Hospital Reina Sofía, Área de Salud de Tudela, Servicio Navarro de Salud—Osasunbidea, 31500 Tudela, Spain; 5 Grupo de Investigación en Deporte y Educación Física para el Desarrollo Personal y Social (GIDEPSO), Department of Specific Didactics, Faculty of Education Sciences and Psychology, University of Córdoba, 14071 Córdoba, Spain; 6 VALFIS Research Group, Institute of Biomedicine (IBIOMED), Faculty of Sciences of Physical Activity and Sports, Universidad de León, 24007 León, Spain; alrof@unileon.es; 7 Valoración del Rendimiento Deportivo, Actividad Física y Salud y Lesiones Deportivas (REDAFLED), Universidad de Valladolid, 42004 Soria, Spain; 8 Neurology Department, Burgos University Hospital, 09006 Burgos, Spain

**Keywords:** energy expenditure, nutrition, metabolic equivalent, indirect calorimetry, activities of daily living, Huntington’s disease

## Abstract

**Background/Objective**: Huntington’s disease (HD) is an autosomal dominant neurodegenerative disorder characterized by motor dysfunction, cognitive impairment, and psychiatric symptoms. As the disease progresses, weight loss, cachexia, and musculoskeletal atrophy are common, reducing quality of life, decreasing their autonomy in their activities of daily living (ADLs), and increasing morbidity and mortality risk. To describe and compare energy expenditure (EE) during ADLs and resting conditions in individuals with HD and healthy controls, and to examine its associations with quality of life, cognitive status, motor function, and functional capacity. **Methods:** A cross-sectional observational study was conducted with 16 people with manifest HD and 10 healthy controls. Participants completed five ADLs: resting, dressing, combing hair, feeding, and walking under laboratory conditions. EE during ADLs was measured using a portable indirect calorimetry system. **Results**: Statistically significant between-group differences in EE were found only during feeding, with individuals with HD showing higher EE than controls (*p* = 0.021). In the exploratory correlation analysis, cognitive status was significantly associated with EE during dressing (*p* = 0.033). **Conclusions**: This exploratory study contributes to the limited evidence on EE during ADLs in adults with HD. The findings suggest that individuals with HD may expend more energy than healthy controls during specific daily activities, particularly feeding. However, these results should be interpreted with caution due to the small sample size and preliminary nature of the study. Larger, multicenter, and longitudinal studies are needed to confirm these findings and determine their clinical relevance.

## 1. Introduction

Huntington’s disease (HD) is an inherited neurodegenerative condition transmitted in an autosomal dominant manner. The disorder is caused by an expanded CAG trinucleotide repeat expansion in the huntingtin gene on chromosome 4. Its clinical presentation includes motor dysfunction [1], cognitive impairment [2] and psychiatric manifestations such as anxiety and depression [3,4]. HD is characterized by the presence of chorea, especially in the intermediate phase, as well as balance disorders, falls, weight loss, difficulty performing voluntary motor tasks, and swallowing problems. Weight loss, often occurring unintentionally, is a common clinical feature of HD and may appear throughout the disease progression. The underlying causes are complex and involve several contributing factors. As a result of these symptoms, people experience functional impairment that, in many cases, leads to total dependence on others, resulting in early institutionalization [5,6].

Malnutrition is considered one of the major clinical concerns in people with HD, and has been associated with increased mortality [7]. People may develop a negative energy balance, in which energy expenditure (EE) exceeds caloric intake, as a consequence of reduced food intake or involuntary physical activity, such as chorea. In this situation, the body may begin to mobilize alternative energy substrates, including adipose tissue and muscle, which can contribute to sarcopenia and cachexia and ultimately accelerate the neurodegenerative process [8,9]. It has been shown that weight loss in patients with cognitive disorders can significantly impair brain function, leading to a worsening of the neurodegenerative process and negatively affecting the progression of the disease. Furthermore, a higher body mass index (BMI) has been associated with a slower rate of disease progression [8,10].

Beyond resting metabolic alterations, the energy cost of routine daily activities may also be clinically relevant in HD. Activities such as feeding, dressing, grooming, and walking are performed repeatedly throughout the day and require the integration of motor coordination, postural control, attention, and executive function. In individuals with HD, chorea, impaired voluntary motor control, reduced muscle strength, fatigue, and cognitive dysfunction may reduce movement efficiency and increase the physiological effort required to complete these apparently low-intensity tasks. Therefore, even small increases in the energy cost of ADLs may have cumulative effects on fatigue, nutritional requirements, weight loss, and functional independence [7,11,12,13].

Increased EE during activities of daily living (ADLs) may also contribute to the high levels of fatigue commonly reported among many neurological patients [14,15,16]. A study that analyzed which symptoms of the disease had the greatest impact on the lives of people with HD concluded that fatigue is one of the most debilitating symptoms [17]. In addition, as the disease progresses, alterations in muscle function and movement disorders may lead to increased EE, which could in turn contribute to a further reduction in physical activity.

Despite the widespread use of the Compendium of Physical Activities [18] in developed countries, researchers have questioned its applicability in populations with neurological conditions. To our knowledge, no study has compared EE during ADLs in individuals with HD. Understanding these metabolic demands may inform dietary modifications, nutritional supplementation, and dietetic counselling, especially when combined with physical activity, as exercise promotes protein synthesis and supports muscle mass maintenance [19].

Previous studies have mainly focused on resting EE, total energy balance, or nutritional status in HD, whereas little is known about the metabolic cost of specific ADLs. This gap limits the ability of clinicians to estimate the real energy demands of everyday functioning and to design nutritional and rehabilitation strategies adapted to the needs of individuals with HD [7,20,21,22].

Therefore, this study aimed to describe and compare EE during ADLs and resting conditions in individuals with HD and healthy controls, and to examine the associations between EE and quality of life, cognitive status, motor function, and functional capacity.

## 2. Materials and Methods

A cross-sectional, observational case–control study was conducted to analyze the differences in EE during ADLs between people with HD and healthy controls.

### 2.1. Participants

The study included a convenience sample of ambulatory individuals with HD recruited from the Neurology Department of BurgosUniversity Hospital; all of whom had a genetically confirmed diagnosed based on the presence of more than 36 CAG repeats in the HTT gene. Symptomatic HD patients were defined by a score higher than 4 on the motor subdomain of the Unified Huntington’s Disease Rating Scale (UHDRS-m) [23], a diagnostic confidence level (DCL) of 4, and the ability to walk with minimal support. The participants were able to understand the task correctly and perform gross ADLs. Healthy controls were recruited from the community and were screened to exclude neurological, cardiovascular, metabolic, pulmonary, musculoskeletal, or endocrine conditions that could affect physical activity or EE. Frequency matching was used to ensure that controls were comparable to participants with HD in terms of age, within one year, and BMI. Controls were not specifically matched for sex, physical activity level, or nutritional status. Participants were excluded if they had a diagnosis of diabetes mellitus, thyroid disorders, other neurodegenerative conditions, cardiac, pulmonary, or musculoskeletal diseases, active cancer, or if they were pregnant or breastfeeding. Individuals receiving medications with potential effects on metabolic or endocrine function were also excluded.

The study was conducted in accordance with Good Clinical Practice guidelines and the ethical principles of the Declaration of Helsinki. It was approved by the Institutional Review Board (University Isabel I and Burgos University Hospital, Certificate number: CEIM-2429)and all participants provided written informed consent before inclusion.

The study was retrospectively registered at ClinicalTrials.gov on 10 February 2022 (NCT05250323), after the beginning of participant recruitment. The study start date was 14 May 2021. At the time of study initiation, the protocol was considered observational and non-interventional, as participants were not assigned to any therapeutic intervention and were only assessed during standardized laboratory-based activities.

### 2.2. Procedures

The study was conducted in the Exercise Physiology, Health, and Quality of Life Laboratory of the University Isabel I (Burgos, Spain). Before the visit, participants were asked to arrive at least 3–5 h after breakfast, to avoid alcohol consumption and nicotine use in the 2 h preceding the visit, and to refrain from moderate or vigorous physical exercise for 24 h before the assessment. These instructions were given to ensure accurate and consistent measurements of EE by standardizing the metabolic state post-breakfast, preventing the influence of alcohol and nicotine on the metabolic rate, and avoiding exercise-induced changes in metabolism. During the initial assessment, demographic, anthropometric, and clinical data were collected. All assessments were performed under standardized laboratory conditions. Before each activity, participants received the same verbal instructions from the research team, and the task was briefly demonstrated when necessary to ensure comprehension. The same testing sequence was used for all participants in order to reduce between-subject variability. Participants were continuously supervised by the research team to ensure safety, particularly during standing tasks and treadmill walking. Short pauses between activities were allowed when needed to avoid excessive fatigue and to ensure that participants could safely continue the protocol.

**Figure 1:**
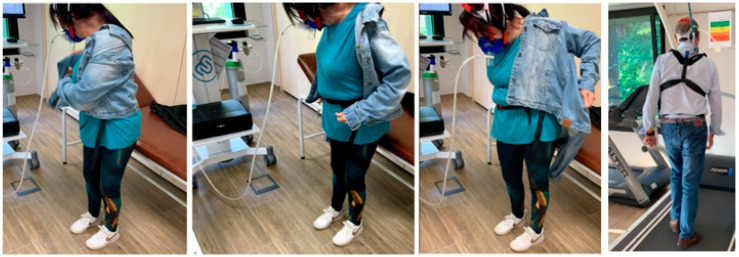
Assessment of energy expenditure during activities of daily living using portable indirect calorimetry. Representative images show a participant performing selected laboratory-based ADLs while wearing the portable indirect calorimetry system.

The participants started the test lying on a stretcher for 10 min while the Medisoft Ergocard (Sorinnes, Belgium), collected resting energy expenditure (REE) data. After the resting assessment, participants performed all the activities detailed in Table 1 without assistance and in the same fixed order: resting, combing hair, feeding, dressing, walking at 3.2 km/h, and walking at 5.2 km/h. The order of the ADL conditions was not randomized in order to standardize the assessment protocol and reduce between-subject variability. The two treadmill walking speeds were selected to represent light-intensity walking and moderate-intensity walking under controlled laboratory conditions. The selected activities represented different functional demands. Feeding was included because it is an essential self-care activity performed several times per day and requires fine motor coordination, bilateral hand use, and visuomotor control. Dressing was selected because it is a complex ADL involving sequencing, balance, bilateral coordination, and motor planning. Combing hair was included as a representative upper-limb grooming task, while walking was selected as a basic mobility activity related to independence and community participation. Resting was used as a reference condition for comparison with active tasks. All activities were performed for 3′30″ to achieve a stable state of gas exchange, except for the rest-activity, which was measured for 10 min. Participants were instructed to avoid holding the front bar when possible, or to use only one hand if needed for safety (Figure 1).

### 2.3. Measures

To measure EE, the Medisoft Ergocard (Medisoft Group, Sorinnes, Belgium), which recorded the respiratory exchange using indirect calorimetry (IC), was used. The device included an adaptive face mask connected to a pneumotachograph and a gas analysis system, which measured oxygen uptake (VO_2_) and carbon dioxide (CO_2_) production, thereby providing respiratory exchange data. Participants breathed through a mask fitted with inspiratory valves, which transmitted O_2_-related information to a computer for subsequent analysis [24]. Before each test, the system was calibrated in accordance with the manufacturer’s guidelines.

Regarding the anthropometric measurements, BMI was calculated using the formula weight (kg)/height (m^2^). The BMI was classified according to the International WHO standards as follows: normal (BMI > 18.5 < 25.0 kg/m^2^), underweight (BMI < 18.5 kg/m^2^), overweight (BMI > 25.0 < 30.0 kg/m^2^), and obese (BMI > 30.0) [25].

Energy and protein intakes and Mediterranean Diet (MeDi) score were assessed based on dietary habits collected through the SUN Food-Frequency Questionnaire and a three-day food diary [23,26]. At baseline, all participants with HD were assessed by a certified neurologist specializing in movement disorders using the UHDRS, including the motor subscale (UHDRS-m), where higher scores reflect more severe motor impairment [23,27]. Disease severity was evaluated through the total functional capacity (TFC) [27], in which higher scores indicate better functional ability. Psychiatric symptom severity was measured using the Problem Behaviours Assessment (PBA) [28,29], with higher scores representing greater symptom severity. Quality of life was assessed with the Short-Form Health Survey 12 (SF-12) [29].

### 2.4. Statistical Analysis

Breath-by-breath data were downloaded from the Medisoft Ergocard metabolic analyzer. Data are presented as mean ± standard deviation (SD). EE was expressed as metabolic equivalents of task (METs). METs were calculated by dividing the measured oxygen uptake during each activity by the standard resting oxygen uptake value of 3.5 mL O_2_·kg^−1^·min^−1^, using the following formula: METs = VO_2_ activity (mL O_2_·kg^−1^·min^−1^)/3.5 mL O_2_·kg^−1^·min^−1^.

The data were then entered into an SPSS version 25 for Windows (SPSS Inc., Chicago, IL, USA) worksheet for statistical analyses. The normality of the data distribution for each variable was verified using the Shapiro–Wilk test. According to the normal distribution of the data, the EE during ADLs was compared between the HD group and controls using an independent-samples *t*-test. Between-group differences were reported using *p*-values, mean differences, 95% confidence intervals (95% CI), and effect sizes. Effect sizes were calculated using Hedges’ g, which is appropriate for small samples. An exploratory correlational analysis was conducted using Spearman’s rho to examine the associations between EE and grip strength, age, SF-12, UHDRS-m, PBA, TFC, and MMSE. Given the small sample size and exploratory nature of the study, *p*-values were not adjusted for multiple comparisons and should therefore be interpreted with caution. Accordingly, statistically significant findings, particularly those from the correlation analyses, should be considered exploratory because of the increased risk of false-positive findings.

The strength of the correlation coefficient (r) was interpreted as follows: values below 0.40 indicated a weak correlation, values between 0.40 and 0.75 indicated a moderate correlation, and values above 0.75 indicated a strong correlation [30].

## 3. Results

The study included 16 individuals with HD and 10 healthy controls. The sociodemographic and clinical characteristics of the participants are presented in Table 2.

No significant between-group differences were observed for age, BMI, FFMI, or MeDi score. However, individuals with HD showed significantly lower grip strength and significantly higher protein intake and total energy intake than controls.

EE during feeding was significantly higher in individuals with HD than in controls (*p* = 0.021). The corresponding mean difference, 95% CI, and effect size are reported in Table 3. Dressing and walking at moderate intensity also showed slightly higher EE in individuals with HD, although these differences did not reach statistical significance.

Table 4 shows the correlation between EE and age, SF-12, UHDRS-m, TFC, PBA, MMSE and grip strength. EE during dressing was significantly correlated with cognitive function; individuals with lower cognitive performance may require more energy to complete this task.

## 4. Discussion

To our knowledge, this is the first study to examine EE during ADLs in individuals with HD using indirect calorimetry. Although EE during ADLs tended to be higher in individuals with HD than in healthy controls, most between-group differences did not reach statistical significance. Feeding was the only activity showing a statistically significant difference, with individuals with HD exhibiting higher EE than controls. Given the small sample size, these findings should be interpreted with caution and considered preliminary.

The higher EE observed during feeding may reflect the greater physiological effort required to perform a fine motor self-care activity in the context of HD. Feeding requires fine motor control, bilateral hand coordination, postural stability, visuomotor integration, and sustained attention. In individuals with HD, involuntary movements, impaired motor planning, reduced movement precision, and difficulties in voluntary motor control may increase the effort required to manipulate utensils and bring food to the mouth. Although the absolute difference in EE observed during feeding was modest, this activity is performed several times per day and may therefore have cumulative clinical relevance, particularly in individuals who are vulnerable to weight loss, malnutrition, fatigue, or reduced functional reserve. However, the present study was not designed to determine whether increased EE during feeding contributes directly to weight loss, fatigue, or functional decline. The trend toward higher EE in other ADLs, although not statistically significant, may suggest a possible increase in metabolic demand during routine tasks in HD. This pattern could be related to the clinical heterogeneity of HD, including differences in motor severity, chorea, cognitive status, muscle strength, nutritional status, and functional capacity. These factors may influence the metabolic cost of daily activities and contribute to interindividual variability in EE. Our findings are consistent with previous research on REE in people with HD, which reported no significant differences in total EE between patients and controls [21].

Gaba et al. [21] compared EE in people with early midstage HD with that of matched controls and found no significant differences between groups. One possible explanation for the lack of significant differences in REE is the reduction in choreic movements under resting conditions. Chorea tends to diminish when patients are lying down or relaxed and disappears completely during sleep. This reduction in involuntary movements at rest may contribute to a metabolic profile comparable to that of healthy individuals, despite the presence of motor symptoms during active states [31].

Our findings are also in line with previous studies conducted in people with other neurological conditions, such as multiple sclerosis (MS) or Parkinson’s disease (PD) [32], which reported similar REE values between people with neurological conditions and healthy controls. By contrast, Doorduijn et al. [33] compared resting EE in people with Alzheimer’s disease (AD) and mild cognitive impairment (MCI) with cognitively healthy controls, and found that individuals with AD and MCI had higher REE than healthy controls, with no interaction with sex. Similarly, Pratley et al. [34] examined sleeping metabolic rate, 24 h sedentary EE, and low spontaneous physical activity in people with HD and reported that sedentary EE was higher in patients with HD than in controls, in proportion to the severity of the movement disorder. Hamilton et al. [35] also found a significant association between weight loss and worsening chorea in patients with HD. Taken together, these studies suggest that EE in HD may vary depending on the context in which it is measured, the disease stage, and the type of activity performed.

Interestingly, when we compared the METs expended during different activities with those reported in a community-based sample from the adult Compendium of Physical Activities (Ainsworth’s Compendium) [18], the MET values observed in people with HD were lower. In particular, significant differences were found for dressing and hair combing. Similar findings have been reported in individuals with intellectual disabilities, in whom EE was lower than the values established in the compendium [36]. Conversely, post-stroke patients have been reported to have higher MET values than those described in Ainsworth’s compendium, which may be explained by the greater oxygen requirements associated with performing ADLs after stroke [37].

Nevertheless, discrepancies in MET estimates between individuals with HD and community-based samples should be interpreted in light of several factors, including body mass, adiposity, age, sex, sarcopenia, movement efficiency, and geographic and environmental conditions. In addition, muscle strength, nutritional intake, physical activity level, cognitive status, and disease-related metabolic changes may influence EE during ADLs. In the present study, BMI, FFMI, and MeDi scores were similar between the HD and control groups. However, individuals with HD showed significantly lower grip strength and significantly higher protein intake and total energy intake than controls. These variables may have influenced the observed differences in EE and should therefore be considered as potential confounding factors. Dietary intake was assessed descriptively but was not included as an adjustment variable because of the small sample size and exploratory nature of the study. Therefore, residual confounding related to nutritional intake, muscle strength, body composition, physical activity level, and disease-related metabolic changes cannot be excluded.

In the present study, EE during dressing was significantly correlated with cognitive function. This association may reflect the cognitive demands involved in planning, sequencing, and executing activities that require attention, memory, and executive functioning. Dressing is a complex task involving multiple steps and decision-making processes, and individuals with cognitive impairment may require more time or effort to complete it, resulting in increased EE during the task. From an occupational perspective, dressing should not be considered solely as a motor task. It also requires body schema, sequencing, bilateral coordination, error detection, problem solving, and sustained attention. Cognitive impairment may therefore lead to less efficient task execution, longer completion time, compensatory movements, and greater physiological effort. These findings suggest that cognitive status should be considered when interpreting EE during ADLs and when designing rehabilitation strategies aimed at maintaining independence in self-care activities. However, this association should be interpreted cautiously because the correlation analysis was exploratory, the sample size was small, and *p*-values were not adjusted for multiple comparisons. The observed correlation may indicate that reduced cognitive performance is associated with less efficient motor execution during daily tasks, even in the absence of overt physical disability [38].

The present findings may have practical implications for the multidisciplinary management of HD. First, measuring EE during ADLs may help clinicians to better estimate the real energy requirements of patients and to adapt nutritional counselling or caloric intake recommendations accordingly. Second, identifying activities with higher energy demands may support the development of individualized occupational therapy and rehabilitation interventions, including energy conservation strategies, task simplification, adaptive equipment, and environmental modifications. For example, in patients who expend more energy during feeding, interventions may include adapted cutlery, postural support, pacing strategies, or caregiver education. Finally, integrating metabolic assessment with neurological, nutritional, and functional evaluations may contribute to a more patient-centred approach aimed at preserving independence, reducing fatigue, and preventing weight loss or frailty.

Our results should be considered in light of several limitations. First, ADLs were performed in a controlled laboratory setting, which may not accurately reflect the conditions in which these activities are typically carried out in everyday life. Although this controlled environment was necessary to standardize measurements, it limits the ecological validity of our findings and may not capture the full range of variability present in free-living conditions. Second, our findings should be interpreted with caution because of the small sample size. The rarity of HD poses significant challenges for recruiting larger cohorts, which affects the generalisability of the results. Additionally, the limited sample size makes it difficult to draw definitive conclusions and increases the potential for data variability. The small sample size also limited statistical power and prevented adjusted analyses. In addition, the correlation analyses were exploratory, and *p*-values were not adjusted for multiple comparisons; therefore, these findings should be interpreted with caution. Third, although controls were frequency-matched by age and BMI, they were not specifically matched for sex, physical activity level, or nutritional status. These variables may influence EE and should therefore be considered as potential confounding factors. Fourth, the order of the ADL conditions was fixed rather than randomized. Although this approach was used to standardize the assessment protocol and reduce between-subject variability, it may have introduced order or fatigue effects. Future studies should consider randomizing or counterbalancing the order of ADL tasks. Furthermore, comparison with previous studies is challenging owing to the scarcity of research focused on energy balance in HD. This lack of evidence highlights the need for further studies to validate and expand our findings.

Despite these limitations, the present study has several strengths. To our knowledge, it is the first study to assess the intensity of ADLs in individuals with HD using direct measurements obtained through indirect calorimetry, which is considered a reference method for measuring EE. This is particularly relevant in a rare genetic condition such as HD, in which little is known about the metabolic cost of daily activities. These findings may inform the design of rehabilitation programmes aimed at reducing gait disturbances, increasing muscle strength, managing fatigue, and ensuring adequate energy intake. Personalized healthcare and patient-centred practice may have a positive impact on both patients and their families.

Future research should validate these preliminary findings in larger and multicenter samples, including individuals at different stages of HD. Longitudinal studies are needed to determine whether increased EE during ADLs is associated with subsequent weight loss, fatigue, functional decline, or reduced quality of life. Future studies should also assess ADLs in home or community-based environments to improve ecological validity and should consider combining indirect calorimetry with wearable sensors to capture both physiological and movement-related variables. In addition, intervention studies are warranted to examine whether nutritional support, occupational therapy, physical rehabilitation, or adaptive strategies can reduce the energy cost of daily activities and improve functional independence.

## 5. Conclusions

In conclusion, this exploratory study contributes to the limited evidence on EE during ADLs in adults with HD. The findings suggest that individuals with HD may expend more energy than healthy controls during specific daily activities, particularly feeding. In addition, lower cognitive performance was associated with higher EE during dressing, which may reflect reduced efficiency when performing more complex self-care tasks. However, these findings should be interpreted with caution due to the small sample size, the exploratory cross-sectional design, and the presence of potential confounding factors, including differences in grip strength and dietary intake between groups. Further studies with larger, multicenter and longitudinal samples are needed to confirm these findings, clarify the factors influencing EE during ADLs, and determine their clinical relevance for functional performance and daily living in individuals with HD.

## Figures and Tables

**Table 1:** Description of activities of daily living.

Activity	Position	Activity Instruction	Equipment
Resting	Supine	Lying supine on a stretcher	Stretcher
Combing hair	Standing	Raise your arm until it touches the top of your head with your hand, with shoulder and elbow flexion, and then lower it until your hand reaches shoulder height.	Hair brush
Feeding	Sitting	Cut a piece of food with the knife, pick it up with the fork, and bring it to the mouth.	Knife, fork, chair
Dressing	Standing	Put on and take off a jacket	Jacket
Walking—light intensity	Standing	Walking on a treadmill at 3.2 km/h with no incline, avoiding handrail support whenever possible	Treadmill
Walking—moderate intensity	Standing	Walking on a treadmill at 5.2 km/h with no incline, avoiding handrail support whenever possible	Treadmill

**Table 2:** Baseline sociodemographic, anthropometric, nutritional, and clinical characteristics of the study participants.

	Controls (n = 10)	Individuals with HD (n = 16)	*p*-Value
Age (years)	51.7 ± 13.61	57 ± 10.07	0.265
Sex, n (%)			
Men	4 (40)	7 (43.8)	
BMI (kg m^−2^)	24.46 ± 4.09	24.29 ± 5.05	0.928
FFMI ^1^	17.17 ± 2.52	16.31 ± 2.07	0.353
Grip strength	30.24 ± 11.04	18.31 ± 7.29	**0.003**
Smokers, n (%)	1 (10)	3 (18.8)	
Protein intake (g/day)	111.23 ± 19.68	139.75 ± 35.02	**0.029**
Total energy intake (kcal/day)	2633.52 ± 534.34	3640.59 ± 858.44	**0.002**
MeDi score	4.7 ± 1.64	4.8 ± 1.74	0.887
UHDRS-m	-	31.67 ± 19.45	-
PBA	-	7.13 ± 11.83	-
SF-12			-
physical component	-	49.83 ± 4.95	-
mental component	-	53.83 ± 10.55	-
MMSE	-	26.47 ± 3.82	-
TFC	-	9.53 ± 3.44	-

BMI: Body Mass Index; ^1^ FFMI: Fat-free Mass Index; MeDi: Mediterranean diet score; UHDRS-m: Unified Huntington’s Disease Rating Scale-motor; PBA: Problems Behavioural Assessment; MMSE: Mini-Mental State Examination; TFC: Total Functional Capacity. Values in bold font indicate statistically significant difference.

**Table 3:** Energy expenditure during activities of daily living measured by indirect calorimetry.

Activity	Controls (n = 10)	Individuals with HD (n = 16)	Mean Difference	EE (METs)		
				95% CI	Hedges’ g	*p*-Value
Resting	1.21 ± 0.28	1.2 ± 0.20	−0.01	−0.20 to 0.18	−0.04	0.812
Combing hair	1.10 ± 0.18	1.23 ± 0.19	0.13	−0.03 to 0.29	0.68	0.112
Feeding	1.20 ± 0.18	1.36 ± 0.20	**0.16**	0.002 to 0.318	**0.80**	**0.021**
Dressing	1.50 ± 0.24	1.72 ± 0.29	0.22	−0.01 to 0.45	0.78	0.058
Walking (light intensity)	2.50 ± 0.46	2.60 ± 0.32	0.10	−0.22 to 0.42	0.26	0.375
Walking (moderate intensity)	3.20 ± 0.56	3.35 ± 0.49	0.15	−0.28 to 0.58	0.28	0.403
Total	1.77 ± 0.28	1.83 ± 0.25	0.06	−0.16 to 0.28	0.22	0.549

Mean differences were calculated as individuals with HD minus controls. Effect sizes are reported as Hedges’ g. CI: confidence interval; EE: energy expenditure; HD: Huntington’s disease; METs: metabolic equivalents of task. Values in bold font indicate statistically significant difference.

**Table 4:** Spearman’s rho correlations between EE during ADLs and clinical variables in individuals with HD.

n = 16		Age	UHDRS-m	TFC	PBA	SF-12 Physical Health	SF-12 Mental Health	MMSE	Grip Strength
**Activity**	Resting	−0.225	0.279	−0.086	−0.073	−0.083	0.319	−0.479	−0.086
	Combing hair	−0.176	−0.416	0.472	−0.013	0.105	−0.028	0.054	0.234
	Feeding	−0.166	−0.003	0.235	−0.162	0.088	0.176	−0.48	0.193
	Dressing	−0.122	0.028	0.275	−0.166	−0.062	0.247	−0.553 *	0.109
	Walking (light intensity)	0.041	0.233	0.257	−0.306	−0.115	0.087	−0.191	0.180
	Walking (moderate intensity)	0.225	0.039	0.222	−0.271	−0.171	0.144	−0.211	0.191
	Total	0.097	0.349	0.248	−0.184	0.02	−0.14	0.103	0.366

* *p* < 0.05. UHDRS-m, Unified Huntington’s Disease Rating Scale (motor); PBA: Problem Behaviours Assessment; SF-12: Short-Form Health Survey 12; MMSE: Mini-Mental State Examination. Values in bold font indicate statistically significant difference.

## Data Availability

The data presented in this study are available upon request from the corresponding author. The data are not publicly available due to privacy or ethical restrictions.

## Abbreviations

The following abbreviations are used in this manuscript:

AD	Alzheimer’s disease
ADLs	Activities of Daily Living
BMI	Body Mass Index
DCL	Diagnostic Confidence Level
EE	Energy Expenditure
FFMI	Fat-Free Mass Index
HD	Huntington’s Disease
IC	Indirect Calorimetry
MCI	Mild Cognitive Impairment
MeDi	Mediterranean Diet
METs	Metabolic Equivalent of Task
MMSE	Mini-Mental State Examination
PBA	Problem Behaviours Assessment
PD	Parkinson’s Disease
REE	Resting Energy Expenditure
SF-12	Short-Form Health Survey 12
TFC	Total Functional Capacity
UHDRS	Unified Huntington’s Disease Rating Scale
UHDRS-m	Unified Huntington’s Disease Rating Scale-motor subscale
